# Mass media and communication interventions to increase HIV testing among gay and other men who have sex with men: Social marketing and visual design component analysis

**DOI:** 10.1177/1363459320954237

**Published:** 2020-09-19

**Authors:** Julie Riddell, Gemma Teal, Paul Flowers, Nicola Boydell, Nicky Coia, Lisa McDaid

**Affiliations:** University of Glasgow, UK; The Glasgow School of Art, UK; University of Strathclyde, UK; University of Edinburgh, UK; NHS Greater Glasgow and Clyde, UK; University of Glasgow, UK; The University of Queensland, Australia

**Keywords:** HIV prevention, HIV testing, men who have sex with men, social marketing

## Abstract

Mass media and communication interventions can play a role in increasing HIV testing among gay, bisexual and other men who have sex with men (GBMSM). Despite the key role of social marketing principles and visual design within intervention development of this type, evidence is limited regarding interventions’ social marketing mix or visual design. As part of a systematic review, intervention content was assessed using social marketing theory and social semiotics. Data were extracted on the nature of the intervention, mode of delivery, use of imagery, content and tone and the eight key characteristics of social marketing. Data were synthesised narratively. Across the 19 included studies, reference to social marketing principles was often superficial. Common design features were identified across the interventions, regardless of effectiveness, including: the use of actors inferred to be GBMSM; use of ‘naked’ and sexually explicit imagery; and the use of text framed as statements or instructions. Our results suggest that effective interventions tended to use multiple modes of delivery, indicating high social marketing complexity. However, this is only part of intervention development, and social marketing principles are key to driving the development process. We identified consistent aspects of intervention design, but were unable to determine whether this is based on evidence of effectiveness or a lack of originality in intervention design. An openness to novel ideas in design and delivery is key to ensuring that evidence-informed interventions are effective for target populations.

## Introduction

Current UK guidelines recommend annual HIV testing for all gay, bisexual and other men who have sex with men (GBMSM), and quarterly testing for men at higher risk (Clutterbuck et al., 2018). Within the current guidelines, high risk includes any unprotected sexual contact with a new partner, diagnosis of a new STI or drug use (in which a detailed sexual history should be taken) (Ross et al., 2014). However, only half of UK GBMSM report annual testing, and less than one quarter defined as ‘at risk for HIV’ test more frequently (McDaid et al., 2016). An estimated 9% of GBMSM were undiagnosed in 2017 (Nash et al., 2018). Delayed diagnosis is associated with poor health outcomes and reduced access to biomedical HIV prevention (Conserve et al., 2017; May et al., 2011; Nakagawa et al., 2012). Pre-exposure prophylaxis (PrEP) is the use of an antiretroviral medication by HIV-negative people to prevent infection and increased availability of PrEP may provide the potential to substantially decrease HIV incidence (Nash et al., 2018). However, it is important to recognise that raising awareness and routine HIV testing will play a key role for those wishing to access PrEP. Increased routine testing plays a key role in reducing undiagnosed and onward HIV infection, and there is a growing body of evidence to suggest that social marketing interventions may be an effective strategy in changing HIV testing behaviour (Carmona and O’Rourke, 2015; McDaid et al., 2019; Olawepo et al., 2018; Wei et al., 2011).

Social marketing can be used in combination with traditional health promotion strategies and focuses on developing activities to change or maintain behaviours in ways that can benefit both the individual and society (Hastings, 2007; Lee and Kotler, 2011). However, we suggest a better understanding of the role of social marketing principles and visual design is required to facilitate development of evidence-informed interventions. Here we use social marketing principles (Hastings, 2007; Lee and Kotler, 2011) and social semiotics (Clarke, 2005; Jewitt and Oyama, 2001; Kress and Van Leeuwen, 1996; Rose, 2016) to further our understanding of HIV testing intervention development.

Social marketing brings together methods from behavioural theory, persuasion psychology and marketing science to design the appropriate delivery and marketing mix (place, price, product and promotion) of health behaviour messages, based on an understanding of how these messages may be interpreted by the viewer (Evans, 2006). Interventions aim to influence individuals to adopt the promoted behaviour or to prompt changes in social norms or existing policies (Firestone et al., 2017).

Semiology is the study of ‘signs’, and offers a variety of analytical tools to deconstruct and interpret the meaning of images (Rose, 2016). Semiology is concerned with the social effects of meaning, revealing power relations and ideological messages inherent in visual representation. Semiology can be used to decode meaning from various visual materials and contexts, in particular it has been extensively applied to study advertising and branding, revealing deep social assumptions and the influence of capitalist ideology (Williamson, 1978). In contrast to the more quantitative approach used in content analysis (Rose, 2016), semiology enables the analyst to dissect images and examine their meaning in relation to the broader cultural context, for example, societal attitudes towards HIV testing generally and amongst GBMSM.

Social semiotics places particular emphasis on the context in which interpretation takes place (Van Leeuwen, 2005), and how images are viewed as part of a communication process or event (Rose, 2016) (e.g. seeing a poster as part of a visit to a GP clinic). Proponents of this approach suggest that the social context of viewing can influence how an image is viewed and interpreted. Three key dimensions relate to the social context of viewing: (1) other imagery that may surround the materials, (2) the social rules for how ‘a spectator should behave in this setting, including whether and how they should look’ (Rose, 2016: 21) and (3) whether the imagery will be viewed in the presence of others, including people who may not be the intended audience (Rose, 2016). Social semiotics also emphasises the importance of multimodal research (Jewitt, 2009) to consider the different ‘modes’ of communication beyond just the image itself, for example, text, music, branding and layout, all of which contribute to how meaning is made.

The current study was part of a systematic review of mass media and communication interventions for HIV testing with GBMSM conducted globally. Here we describe the social marketing and visual component analysis, conducted alongside a review of effectiveness and behaviour change component analysis, reported elsewhere (Flowers et al., 2019; McDaid et al., 2019). The following paper aims to describe the social marketing and visual design components commonly found in HIV testing campaigns and furthermore to determine what social marketing and visual design components are linked to effectiveness (evidence of clear behaviour change in the desired direction, i.e. an increase in HIV testing), to inform further work to develop an evidence-informed and theoretically based social marketing intervention.

## Methods

### The review

Full details of the systematic review are reported elsewhere (McDaid et al., 2019), however we have included a summary below.

### Search strategy

CINAHL, Embase, Medline, PsychInfo and Web of Science were searched for peer-reviewed studies published between 1st January 2009 and 15th November 2016, using detailed search strategies and standard MESH terms for HIV, GBMSM and social marketing/mass media interventions. Previous reviews suggest that not all social marketing interventions will be labelled as such (Stead et al., 2007), therefore we expanded the inclusion criteria to avoid excluding relevant studies. An example of the search strategy applied to Medline is presented in Supplemental File 1. In addition to database searches, reference lists of included articles were searched manually. A maximum of three requests were made to study authors for visual materials (videos, posters, etc.).

### Study selection

Peer-reviewed studies with visual materials in English, Spanish or Italian were included as translation services were available. Studies in which GBMSM constituted at least one third of the sample, included interventions that sought to change behaviour through non-interactive, visual or auditory means and included HIV testing as an outcome were included. Studies included within a previous Cochrane review were included where relevant (Guy et al., 2009; McOwan et al., 2002).

### Data extraction

Structured data extraction tools were developed to capture required information. Data extraction was completed by one author (JR) with a 10% sample validated by another (NB). Discrepancies were resolved through consensus or discussion with the wider team.

#### Social marketing principles and complexity

Data were extracted, from intervention descriptions and visual materials, on the nature of intervention, mode of delivery, use of imagery, content and tone of the visual materials, using the following eight key social marketing principles as a guide (Hastings, 2007).

(1) Behaviour change focus
(2) Theoretical framework employed in intervention design
(3) Insight driven
(4) Customer orientation (e.g. consumer research and pretesting)
(5) Segmentation and targeting
(6) Motivational exchange
(7) Competition (i.e. considers appeal of competing behaviours and uses strategies to overcome these
(8) Marketing mix (product, place, promotion and price)

In this study, standard social marketing definitions of product, place, promotion and price were used (Hastings, 2007). Product reflects (1) the underlying values and benefits associated with performing the target behaviour (HIV testing), (2) the actual performance of the target behaviour (HIV testing) and (3) the ways in which value is added to enrich the experience of behaviour (HIV testing). Price includes instrumental costs (e.g. costs of testing kits) which may be endured during performance of the behaviour (HIV testing) and costs tied to negative emotions (e.g. fear of negative results). Place captures distribution channels where viewers acquire information related to performance of the behaviour, including networks and physical spaces. Promotion identifies key messages, delivery modes and messengers needed to inform about target behaviour and inspire action (Lee and Kotler, 2011).

Characteristics of the social marketing mix of each of the included studies were reviewed and categorised as representing low or high intervention complexity by one author (LMcD) and checked for consistency by another (JR). The social marketing mix (product, place, promotion and price) were determined to contain 11 components (Table 1). Low complexity was defined as the inclusion of single elements or absences for each, while high complexity was defined as the inclusion of multiple elements (e.g. the use of a single image across a campaign was defined as low complexity and the use of multiple different images was defined as high complexity). Interventions considered to meet at least seven of the 11 criteria for complexity (Table 1) were defined as having high overall social marketing complexity.

**Table 1.** Defining overall complexity of intervention social marketing mix.

Social marketing mix		Low complexity	High complexity
Product	Pre-testing	None	User informed
	Provider^†^	Static	Interactive
	Content	Single	Multiple
	Frequency and duration	One off/short	Long term
	Imagery	Single	Multiple
	Tone	Single	Multiple
Promotion	Segmentation and targeting	All GBMSM	Tailored
	Modes of delivery	Single	Multiple
Place	Settings	Single	Multiple
Price	Motivation	Absent	Present
	Competition	None/single	Multiple

† Provider – static = delivered by two dimensional media only, that is, posters/leaflets; interactive = delivered by varied media requiring participants to engage with content, that is, banner ad linking to online video.

#### Visual analysis

Materials were coded using 28 descriptive dimensions (Table 2) based on Kress and van Leeuwen’s social semiotic approach (Jewitt and Oyama, 2001). This provides a framework for visual analysis that supports detailed and systematic description and interpretation of the meaning of visual materials (Kress and Van Leeuwen, 1996). For the current study, this framework was adapted to include descriptors relating to social context of viewing and consideration of the intended or unintended viewers of the visual material (where defined in intervention descriptions) (Clarke, 2005; Rose, 2016) and technical aspects related to production (i.e. medium of the image and mode of delivery). Coders recorded detailed descriptions of the content of images using the framework as a guide. Finally, we considered the overall effect of multimodal materials, including consistency of the combined modes, originality of materials and resulting tone. These approaches are suited to the analysis of visual materials using still images and were adapted for analysis of videos to consider dimensions such as duration and sound.

**Table 2.** Visual analysis assessment criteria.

Visual components		Examples
(1) Technical	Type of intervention	Poster, leaflet, video
	Medium (could be more than one)	Photo, video, diagram, illustration, 3D letter
	Effects	Lens flare, fish eye, flash lighting, special photo processing
(2) Reading the visual	Actor’s appearance	
	Setting/Environment	
	Props/Objects	
	Form of representation	Narrative (action, transactions, mental/verbal processes)
		Conceptual (classificational, analytical, symbolism)
	Contact	Demand (e.g. direct eye contact, offer of information, services or goods)
	Social distance	Intimate (close up), medium (social), impersonal (distance)
	Point of view	Engagement, involvement, detachment
		Viewer power, equality, representation power
	Compositional (salience)	Information value, framing, colour, focus, texture, scale
	Modality	High/medium/low level of truth to image
(3) What supports the visual?	Text	Content, form (e.g. questions, speech, instructions), font (colour, tone, weight)
	Logos	Relative size, location, type of organisation, recognisable by audience
	Audio	Music, sound effects, speech?
(4) Social context of viewing	Location of materials	Clinic, public billboard, gay scene venue
	References to visual culture	Soap opera style
	Societal norms, stereotypes, stigmas, controversies at play	
(5) Overall - combination	Intended/unintended audiences	
	Originality	Unique, surprising
	Provocation	Fear, humour, warmth, irritation, sexual arousal, incongruity, ambiguity
	Consistency of messages	
	Tone	

The extracted data were analysed for patterns in representation and ‘outliers’, here outliers refers to those which significantly differed from the design elements consistently evident in other interventions. These patterns and outliers were then compared to the relative effectiveness of interventions.

Data extraction was completed by one author (JR), a 10% sample was also coded by another author (NB) and discrepancies were identified. Discrepancies were then resolved through consensus or through discussion with a third author (GT).

#### Patterning of effectiveness

Studies were categorised in terms of relative effectiveness under the following five categories:

(1) Negative effect reported (i.e. decrease in uptake of HIV testing)
(2) No reported evidence of positive or negative effects
(3) Reported effect on the antecedent of behaviour (e.g. intentions to test or knowledge)
(4) Indicative of some positive desired behaviour change
(5) Indicative of clear behaviour change in desired direction

Social marketing complexity and visual components were then mapped against effectiveness to identify the associated intervention characteristics.

## Results

Nineteen articles were included in the review, focusing on 22 interventions (McDaid et al., 2019). Seventy items of visual materials were obtained for analysis, with the majority supplied directly by study authors (*n* = 55). Materials included in the visual analysis covered 14 interventions, ranging from 1 to 22 items per intervention (Table 3).

**Table 3.** Number of interventions materials and location of intervention.

Intervention name	Number of materials provided	Location of intervention	
		GBMSM community	Mainstream setting
Drama down under (Pedrana et al., Wilkinson et al.) Victoria, Australia	22	Yes	Yes
I’m testing (James) England, UK	16	Yes	Yes
Make your position clear (Flowers et al.) Scotland, UK	6	Yes	Yes
Hottest at the start (Gilbert et al.) British Columbia, Canada	6	Yes	No
Gimmie 5 minute (McOwan et al.) London, UK	4	Yes	No
*Not named* (Brady et al.) United Kindom	3	Yes	Unclear
The morning after (Hirshfield et al., Chiasson et al.) USA	3	Yes	No
Check it out (Guy et al.) Victoria, Australia	2	Yes	Yes
Get Tested with Via Libre (Blas et al.) Lima, Peru	2	Yes	No
United against AIDS (Prati et al.) Italy	2	No	Yes
Talking about HIV (Hirschfield et al.) USA	1	Yes	No
POCT (West) England, UK	1	Yes	Unclear
Crowdsourcing video (Tang et al.) China	1	Unclear	Unclear
Health Marketing intervention video (Tang et al.) China	1	Unclear	Unclear
HIV wake up intervention (Hilliam et al.) Scotland, Uk	0	Yes	Yes
I did it (Hickson et al.) England, UK	0	Unclear	Unclear
Clever dick/smart arse (Hickson et al.) England, UK	0	Unclear	Unclear
Count me in (Hickson et al.) England, UK	0	Unclear	Unclear
You know different (Thackerey et al.) USA	0	Unclear	Unclear
*Not named* (Erausquin et al.) California, USA	0	Yes	No
What are you waiting for (Gilbert et al.) British Columbia, Canada	0	Yes	No
Tu Amigo Pepe (Solorio et al.) Seattle, USA	1 (website only provided)	Yes	Yes
Total	70		

### Social marketing component analysis

The social marketing component analysis covers all 22 interventions, with data extracted from intervention descriptions rather than visual materials, with the exception of marketing mix, which was also observed in visual materials. Of the 19 included studies, ten explicitly discussed some of the eight social marketing principles, but not all (Flowers et al., 2013b; Gilbert et al., 2013; Guy et al., 2009; Hickson et al., 2015; Hilliam et al., 2011; Hirshfield et al., 2012; Pedrana et al., 2012; Solorio et al., 2016; Thackeray et al., 2011; Wilkinson et al., 2016), while just two referenced all eight (Blas et al., 2010; Thackeray et al., 2011). Although Blas et al. (2010) made reference to all eight principles, social marketing theoretical principles were not explicitly discussed within the paper. Reflection on social marketing principles within intervention descriptions was often superficial and basic. The following findings are presented within subsections relating to the social marketing principles.

### Theory and behavioural goals

Although most studies included some discussion of theoretical constructs, only six reported applying a formal theoretical framework. Nevertheless, we acknowledge that theoretical constructs were often implicit within materials rather than explicitly discussed within intervention descriptions.

In terms of behavioural goals, twelve studies focused on changing HIV testing (Chiasson et al., 2009; Erausquin et al., 2009; Guy et al., 2009; Hickson et al., 2015; Hilliam et al., 2011; Hirshfield et al., 2012; James, 2015; McOwan et al., 2002; Pedrana et al., 2012; Prati et al., 2016; Thackeray et al., 2011; West et al., 2015), two focused on increasing regular HIV testing (Flowers et al., 2013b; Wilkinson et al., 2016) and one focused on first time testing (Tang et al., 2016). Two studies sought to increase HIV testing by raising awareness of new testing technologies (Brady et al., 2014; Gilbert et al., 2013) and two focused on antecedents of behaviour change (e.g. intention to test) (Blas et al., 2010; Solorio et al., 2016). How studies sought to achieve increases in testing was not often clearly defined.

### Insight and customer orientation

Key to effective social marketing interventions is that they are insight-driven and grounded in sound consumer research (Hastings, 2007). Only eight studies reported some form of developmental research or pre-testing of visual materials (Blas et al., 2010; Erausquin et al., 2009; Flowers et al., 2013b; Guy et al., 2009; Pedrana et al., 2012; Solorio et al., 2016; Thackeray et al., 2011; Wilkinson et al., 2016).

### Segmentation/targeting

Segmentation and targeting are important components of social marketing, recognising the diverse audiences who could or should be exposed to the intervention (Hastings, 2007). Although GBMSM are not a homogenous group, just two studies referred to segmentation and development of materials specifically on the basis of sexual identity (i.e. gay-identified and non-gay identified GBMSM) (Blas et al., 2010; Solorio et al., 2016). Segmentation based on demographics (age, location and ethnicity) were reported to a limited extent, with five targeting ‘younger’ age groups (Erausquin et al., 2009; Guy et al., 2009; McOwan et al., 2002; Solorio et al., 2016; Thackeray et al., 2011) and seven segmented by ethnicity (Erausquin et al., 2009; Guy et al., 2009; James, 2015; McOwan et al., 2002; Prati et al., 2016; Solorio et al., 2016; Thackeray et al., 2011). Ten studies reported interventions delivered within a specific city or region (Blas et al., 2010; Erausquin et al., 2009; Flowers et al., 2013b; Guy et al., 2009; McOwan et al., 2002; Pedrana et al., 2012; Solorio et al., 2016; Thackeray et al., 2011; West et al., 2015; Wilkinson et al., 2016) with the remainder identified as national interventions. Segmentation by risk behaviours or previous testing was rare. Only one study reported targeting men who had never tested (Tang et al., 2016), another addressed sexual risk behaviours (Hirshfield et al., 2012), however, these were identified as part of the content of visual materials rather than as a component of segmentation. None of the studies reported using motivations for testing (or not) as a way to target interventions.

### Motivation and competition

Just one study reported an exchange analysis, giving full consideration of disincentives for testing in terms of risk of judgement/shame and having to change behaviour, compared with the incentive of a sense of responsibility and HIV status knowledge (Thackeray et al., 2011). Other studies implied different issues that may be disincentives (e.g. stigma) but failed to present a clear description or analysis of the perceived and actual costs of interventions compared with perceived and actual benefits. Two studies commented on the time involved in engaging with the intervention in terms of disincentives (Chiasson et al., 2009; Erausquin et al., 2009).

### Marketing mix

The marketing mix includes four aspects (*price, product, place* and *promotion*) and the extent to which these were described by the studies included in the review is shown in Supplemental File 2.

Price, the potential costs of a desired behaviour that users have to overcome, can present both internal and external barriers that compete with the appeal of an intervention (Hastings, 2007). While eleven studies reported strategies to overcome competing behaviours and costs (Blas et al., 2010; Chiasson et al., 2009; Guy et al., 2009; Hickson et al., 2015; Hilliam et al., 2011; Hirshfield et al., 2012; Prati et al., 2016; Solorio et al., 2016; Thackeray et al., 2011; West et al., 2015; Wilkinson et al., 2016), the ways in which these strategies were incorporated into interventions were often unclear with only one study providing a detailed exchange analysis (Thackeray et al., 2011).

Product can be defined as the nature and aim of the intervention, the use of branding, frequency/duration, intensity and the content, tone and imagery used. Seventeen studies reported use of an intervention name, brand or logo (Blas et al., 2010; Brady et al., 2014; Chiasson et al., 2009; Flowers et al., 2013b; Gilbert et al., 2013; Guy et al., 2009; Hickson et al., 2015; Hilliam et al., 2011; Hirshfield et al., 2012; James, 2015; McOwan et al., 2002; Pedrana et al., 2012; Prati et al., 2016; Solorio et al., 2016; Tang et al., 2016; West et al., 2015; Wilkinson et al., 2016). Interventions were delivered for up to 14 months, although few reported on intensity (e.g. length of time or number of times potential users might engage with visual materials). While interventions used a variety of imagery, the majority used photographs (*n* = 9) or video footage (*n* = 7) with only one using a cartoon animation (Tang et al., 2016).

**Figure 1.**
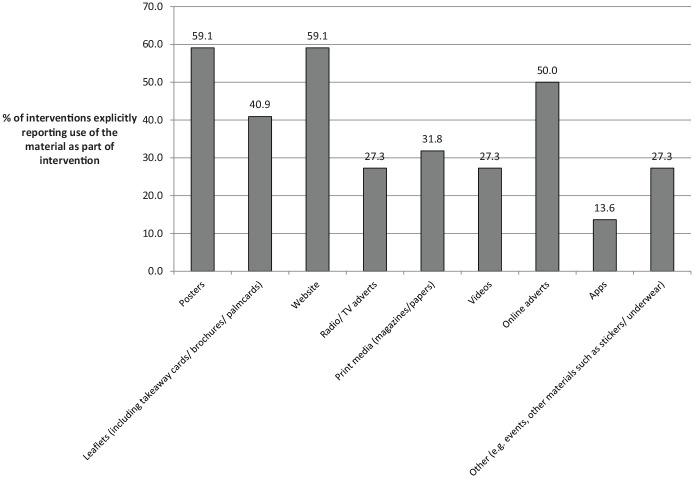
Materials explicitly stated as used in interventions (*n* = 22)*. *Note some interventions used more than one type of material.

Promotion describes modes of delivery (e.g. posters, radio, TV, YouTube or other materials), with multiple modes generally employed within each intervention (Figure 1). In terms of place, the majority reported that interventions were delivered online (*n* = 13), in gay venues (*n* = 8) or other community settings (*n* = 7). Other settings included radio (*n* = 3), newspapers/magazines (*n* = 3) or in clinics (*n* = 1).

### Effectiveness and social marketing mix

Interventions meeting at least seven of the 11 criteria for high complexity in the use of social marketing mix were defined as having high overall complexity. Complexity was mapped against effectiveness (Table 4). A greater proportion of studies reporting results indicative of behaviour change in the desired direction scored high for overall complexity than other grouped measures of effectiveness. Even so, three of the ineffective interventions were also scored as highly complex, although one of these interventions was found to be effective within another study (Pedrana et al., 2012; Wilkinson et al., 2016). Customer orientation and segmentation were key aspects of most of the highly complex (and effective) interventions. Despite Prati et al. (2016)^1^ being considered a highly complex intervention, it did not appear to use appropriate segmentation, nor report customer orientation or pre-testing of the visual materials and was found to be ineffective.

**Table 4.** Social marketing complexity and intervention effectiveness.

Study	Intervention had no effect	Intervention had an effect on the antecedent of behaviour (e.g. intentions to test or knowledge)	Indicative of some positive desired behaviour change	Indicative of clear behaviour change in desired direction
Blas et al. (2010)^†^ Lima, Peru			Low overall complexity	
Brady et al. (2014)^†^ England, UK			Low overall complexity	
Chiasson et al. (2009)^†^ United States of America	Low overall complexity			
Erausquin et al. (2009) Los Angeles County, United States of America			High overall complexity	
Flowers et al. (2013)^†^ Glasgow, Scotland				High overall complexity
Gilbert et al. (2013)^†^ British Columbia, Canada				High overall complexity
Guy et al. (2009)^†^ Victoria, Australia	High overall complexity			
Hickson et al. (2015) England, UK			Low overall complexity	
Hilliam et al. (2011) Scotland, UK				Low overall complexity
Hirshfield et al. (2012)^†^ United States of America	Low overall complexity			
James (2015)^†^, England, UK			High overall complexity	
McOwan et al. (2002)^†^, England, UK				High overall complexity
Pedrana et al. (2012)^†^ Victoria, Australia				High overall complexity
Prati et al. (2016)^†^ Italy	High overall complexity			
Solorio et al. (2016), Seattle, USA		High overall complexity		
Tang et al. (2016)^†^, China				Low overall complexity
Thackeray et al. (2011) United States of America				High overall complexity
West et al. (2015)^†^ England, UK		Low overall complexity		
Wilkinson et al. (2016)^†^ Victoria, Australia	High overall complexity			

† Included in visual analysis.

### Visual design analysis

Visual materials were available for 14 interventions (Table 3), with 70 individual items sourced. The findings for the visual analysis relate to these 14 interventions, with the exception of findings relating to social context, which were extracted from intervention descriptions and therefore covers all 22 interventions (Supplemental File 3).

### Appearance and compositional aspects of the actor(s), settings and props

Within a visual, the ‘actor’ is the ‘doer’ of the action (Jewitt and Oyama, 2004); all but one study (West et al., 2015) featured actors who could be seen as representative of the target audience (GBMSM). Two interventions used images of simulated sexual positions to explicitly identify actors as GBMSM (Flowers et al., 2013b; Guy et al., 2009), one featured an out gay celebrity (James, 2015) and five videos explicitly used narrative or dialogue to communicate actors’ sexual orientation. One intervention appeared to use a variety of stereotypical visual clues to identify the actor as GBMSM, for example, exaggerated or overly dramatic expressions (Pedrana et al., 2012; Wilkinson et al., 2016). Actors were generally coded as more attractive than average in the majority of interventions.

Only three visual materials did not include images of people; these instead featured a rumpled bed, a pair of cockerels and text as a graphic element (Brady et al., 2014). Nine of the interventions featured the use of naked or semi-naked actors, likely intended to symbolise sex and convey the subject matter. All of the interventions could be defined as using narrative forms of representation rather than conceptual, in that they intended to depict the actor doing something (e.g. showing someone testing or engaging in sex) (Jewitt and Oyama, 2004).

Interventions involving videos often chose to feature testing procedures, using facial expressions and body language to depict staff as friendly and non-judgemental. Regardless of the mode of delivery, materials were generally composed so that actors were positioned centrally, emphasising the actor. The majority of interventions placed the actors in decontextualized settings and were either staged or dramatised, which reduced the truth-value of the visual. For example, one intervention digitally distorted the image of the actor’s body (Pedrana et al., 2012), explicitly referencing anal sex and drawing attention to the actors’ genitals. In this intervention, lighting and digital enhancements were used, reminiscent of commercial underwear adverts.^2^ Another intervention (McOwan et al., 2002) did the opposite, presenting one of the actors with uneven skin and visible spots, potentially to signal normality and make the actor more ‘relatable’ to target younger viewers.

### Social position of the viewer

The relative position of the viewer and image, contact and point of view can be used to infer meaning. Interventions generally used ‘medium’ (or ‘waist’ shots) or close ups (Guy et al., 2009; Hirshfield et al., 2012) to suggest a relationship with viewers that was more social or intimate. Direct eye contact was used within nine of the interventions, engaging viewers through facial expressions described as flirtatious (Pedrana et al., 2012), intense and sexually confident (McOwan et al., 2002), happy and reassuring (Brady et al., 2014; James, 2015), with the remainder described as serious. All interventions placed actors at viewers’ eye-level to create an equal power dynamic.

### Supporting the visual

**Figure 2.**
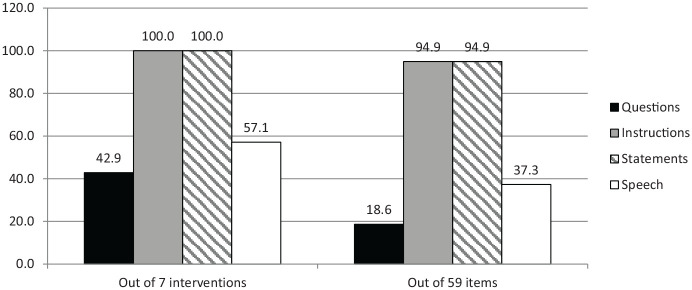
Form of text used within interventions (*n* = 7) and within materials (*n* = 59) that used text in images*. *Note some materials and interventions used more than one aspect.

All but one intervention featured limited amounts of text, most frequently phrased as instructions or statements (see Figure 2). Interventions generally used eye-catching headlines in bold fonts, with longer sub-headings conveying key messages. However, one intervention used a different approach; a main headline (intervention slogan) in large bold font printed over the male actor’s bare chest with three columns of smaller text providing a significantly larger volume of detailed information (McOwan et al., 2002).

### Social context of viewing

In relation to the available information concerning the social context of viewing, 15 of the interventions (Blas et al., 2010; Brady et al., 2014; Chiasson et al., 2009; Erausquin et al., 2009; Flowers et al., 2013b; Gilbert et al., 2013; Guy et al., 2009; Hilliam et al., 2011; Hirshfield et al., 2012; James, 2015; McOwan et al., 2002; Pedrana et al., 2012; Solorio et al., 2016; West et al., 2015; Wilkinson et al., 2016) reported placement in GBMSM community locations (e.g. gay friendly venues) while eight (Flowers et al., 2013b; Guy et al., 2009; Hilliam et al., 2011; James, 2015; Pedrana et al., 2012; Prati et al., 2016; Solorio et al., 2016; Wilkinson et al., 2016) reported mainstream locations (e.g. Public transport/bus stop posters) (Table 3). Seven of the interventions were presented in both (Flowers et al., 2013b; Guy et al., 2009; Hilliam et al., 2011; James, 2015; Pedrana et al., 2012; Solorio et al., 2016; Wilkinson et al., 2016), while only one intervention was designed to be run only in mainstream locations (Prati et al., 2016). Three studies did not provide clear information about the locations they were delivered in (Hickson et al., 2015; Tang et al., 2016; Thackeray et al., 2011).

### Combined effects of visual design elements

Visually, the image tone could be interpreted as: light-hearted, humorous, sexually arousing, positive, reassuring, serious, informative and emotive, although this was subjective. Given the important role that fear of a positive test result plays as a barrier to HIV testing (Deblonde et al., 2010; Flowers et al., 2013a; Lui et al., 2018), it is perhaps surprising that none of the interventions directly used fear, although we would suggest that it was implied where real or dramatised experiences were depicted.

Interventions were generally considered consistent across multiple modalities, that is, imagery and accompany text were aligned in tone and content. Only one intervention was highlighted as having contradictory modes, combining a cartoon-style animation, which could be described as childlike and light-hearted, with serious text (Tang et al., 2016).

### Effectiveness and interventions’ visual design

As visual materials were only obtained for 14 interventions, the results in this section are limited to these (Table 4). Four studies reported results suggesting clear behaviour change (Flowers et al., 2013b; Gilbert et al., 2013; McOwan et al., 2002; Tang et al., 2016) (Table 4), three reported results suggesting some positive change (Blas et al., 2010; Brady et al., 2014; James, 2015) and one reported an effect on the antecedents of behaviour (West et al., 2015). Four studies reported no effect (Chiasson et al., 2009; Guy et al., 2009; Hirshfield et al., 2012; Prati et al., 2016). The remaining intervention had contradictory evaluations, with one study reporting clear behaviour change (Pedrana et al., 2012) while a more recent study, which adopted different analytical techniques and timescales, reported no effect (Wilkinson et al., 2016). Patterning of effectiveness by intervention visual design was complex, with commonalities noted across all levels of effectiveness.

## Discussion

The current paper is part of a series of papers presenting findings from a systematic review exploring evidence related to mass media and communication interventions to increase HIV testing among GBMSM. More detailed analysis relating to intervention effectiveness (McDaid et al., 2019), the active content of interventions (Flowers et al., 2019) and affect used within interventions (Langdridge et al., 2020) can be found elsewhere.

The current analyses employed rigorous data extraction tools to attempt to identify the common social marketing principles and visual design elements associated with effectiveness across a range of HIV testing campaigns. Our findings suggest that there are key features used across campaigns – standard visual content, high complexity of intervention and key messages – which form the basis of most HIV testing interventions.

As previously stated, the current findings suggest that none of the interventions involved in the study explicitly attempted to address fear of a positive HIV test result, which is surprising given previous evidence suggesting that fear of the result may result in avoidance of testing (Deblonde et al., 2010; Flowers et al., 2013a; Lui et al., 2018). Whilst the current findings do consider the overall tone of the content, a more detailed discussion of affect within these interventions can be found in Langdridge et al. (2020).

**Figure 3.**
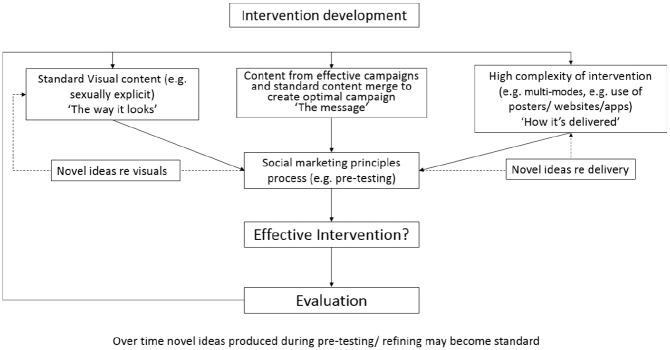
Process of developing and evaluating interventions.

It is important to recognise the key role that social marketing principles play in guiding the processes used to develop effective interventions. The use of formative or pre-testing sessions and meaningful evaluations of intervention acceptability and adherence can not only confirm acceptability of potential interventions but may also serve to introduce novel ideas regarding visuals or delivery methods which may be more effective within the target population (see Figure 3).

Despite the important role that pre-testing can play in intervention development, and in line with previous reviews (Noar et al., 2007), our study found very few of the studies reported formative or pre-testing sessions, which in turn limits our understanding of how key intervention messages originated and were adapted to appeal to the target population and cultural context. Included studies that did report pre-testing employed focus groups to identify key messages, for example, around information provision/addressing reasons for testing (or not), or the practicalities of the intervention (e.g. use of subtitles in videos/leaflets/posters, etc) (Blas et al., 2010; Erausquin et al., 2009; Flowers et al., 2013b; Guy et al., 2009; Pedrana et al., 2012; Solorio et al., 2016; Thackeray et al., 2011; Wilkinson et al., 2016). However, these studies still presented limited information as to the decision-making processes related to the origin of key intervention messages. Few studies referred to the use of the pre-testing sessions in terms of specifying or selecting visual designs, with those that did providing very limited information regarding the selection of images for initial consultation.

Without information related to pre-testing, we question whether the consistent use of sexualised imagery reflects their effectiveness within the target population or simply because they have become normalised in interventions within GBMSM populations. Similarly, we are unclear if consistent use of sexualised imagery has resulted in viewers becoming desensitised, leading to the use of increasingly more explicit imagery in an attempt to attract attention in a way not typically seen in other populations

The wider social context is also important in interpreting visual materials; societal norms, stereotypes, stigmas and controversies may influence how visuals are viewed. Within the current study many of the interventions were drawn from outside the UK, thus it was difficult for us to comment on these nuanced contextual aspects. However, two of the interventions could be interpreted as reinforcing stereotyped norms of GBMSM as ‘promiscuous’ and sexual ‘risk-takers’ (Flowers et al., 2013b; Gilbert et al., 2013) with several visuals depicting sexual encounters in public places (Gilbert et al., 2013). Although, it is important to note that the limited information regarding pre-testing, or on how representative GBMSM involved in this might be, means we are unable to determine the level of involvement GBMSM may have had in the creation of these images and thus how culturally specific these messages were.

Drumhiller et al. (2018) suggested that some intervention recipients may prefer images that are more broadly relatable, less stereotypical and not identifiable as GBMSM, to avoid stigmatising GBMSM as solely affected by HIV. As more extensive formative research is associated with a greater likelihood of impact (Stead et al., 2007), we would suggest that this should be a key part of the development process and should be clearly described when reporting on the intervention. Such formative research should relate not only to the positive, but also to potentially harmful effects that images may have on the viewers (Drumhiller et al., 2018).

However, we recognise that the absence of process evaluations or intervention manuals within our review limits our understanding of the intervention development; indeed these principles may have been adhered to within the development process but not explicitly stated within the literature identified. As a result, we suggest that future interventions need to be explicit regarding their use of social marketing principles and the process of intervention development.

Finally, a lack of information regarding comparable interventions both within the country of origin and in terms of the context of viewing, including other materials adjacent to the intervention, results in difficulties assessing the originality of the interventions. Again, information relating to development and pre-testing is vital in our understanding of how interventions are developed in consultation with the local target population in order to fully appreciate the specific cultural context.

### Strengths and limitations

We decided to include all available visual materials on the basis that in addition to decoding the meaning of visual materials, we also sought to identify patterns of effectiveness. This differs from the standard approach used in semiotic studies, which presents detailed case studies of relatively few images deemed conceptually interesting (Rose, 2016).

Our limited understanding of the social context in which images were viewed may have limited our ability to understand the role of other factors on effectiveness. The researchers who led data extraction, coding and interpretation were cisgender, heterosexual women living in the UK and as such not the intended target audience. This meant identifying specific cultural references was challenging, unless explicitly discussed in intervention descriptions. For example, where an intervention featured an unfamiliar celebrity or logo it was not possible to draw conclusions about the meaning attributed to the inclusion of the person/logo, unless explicitly stated. Studies rarely provided detailed information or images to describe how visual materials were presented within each setting, and what other images were adjacent. For example, sexualised, or sexually explicit images, if presented within a context in which these images were expected, may ‘blend in’ with other interventions, potentially reducing their impact.

In the current study, we also recognise that the researchers may have misinterpreted visual images based on their own assumptions and sociocultural norms, particularly when conducting visual analysis of subjective elements, for example, relative attractiveness of actors. While data extraction tools were developed in such a way as to reduce the impact of this on the current findings, these limitations in analysing interventions from other countries should be recognised in terms of analysis and applicability.

Finally, whilst intervention descriptions often lacked detail, they did, at times, contain information that may have influenced our perception of visual materials, even at a subconscious level. For example, where descriptions detailed targeting specific groups, researchers may subconsciously code visual materials to interpret actor choices as more or less representative of the target audience in a way they might not have without this information. To aid transparency of coding, visual materials were coded separately from intervention descriptions, and visual aspects were only coded if they could be ‘read’ directly from the visual material. We acknowledge this limitation both in coding, and the resulting analysis and interpretation.

While we acknowledge limitations within the current study, a follow up study (Flowers et al., Forthcoming; Teal et al., Forthcoming) invited GBMSM to take part in workshops where we presented a subset of these visual materials in order to ascertain their views on the images. Views gathered during these workshops validated and enriched the above findings within the target audience, although we recognise that some cultural nuances may still have been missed. For example, men generally agreed with the interpretation that images were designed in such a way that GBMSM were the obvious target of the intervention, with many of the men commenting on the sexually explicit nature of the images (both positively and negatively).

## Conclusion

The current study has provided insights into imagery consistently used within social marketing interventions for HIV testing. Our results have identified aspects of visual design that appear to be consistent across the included interventions and suggest the consistent use of high complexity within effective interventions. However, we would suggest that this is only part of intervention development, and that social marketing principles are key to driving the development process, in particular the use of pre-testing of interventions.

Intervention developers may wish to use components identified as ‘standard’ content or those specifically linked to effective interventions as a basis for intervention development, however relying solely on existing literature may prohibit the development of novel interventions that may prove more effective. Thus, the key to engaging target populations in future interventions is ensuring that those developing interventions are open to new ways of presenting messages which are pre-tested or developed with the target audience.

Within the current study, we found intervention descriptions rarely explicitly discussed social marketing principles, supplied images in situ or provided detailed descriptions of the cultural, political and services context within which the intervention was delivered. In order for intervention developers to learn from previous interventions, we believe that this information is vital. While we recognise that this may reflect a gap in reporting, detailed intervention descriptions, which explicitly discuss social marketing principles such as motivation and competition and are explicit around decisions relating to intervention material development (e.g. choice of image) are essential for future development.

Very few interventions reported on unintended consequences or negative consequences of interventions. Effective intervention development requires an understanding of both what works, and where interventions may have either no effect or negative consequences, for example, discouraging testing.

Finally, while our findings relate specifically to HIV testing interventions, we feel that the broad concepts included within Figure 3 are applicable across all intervention design. An openness to novel ideas relating to visual aspects and delivery is key to ensuring that interventions are effective and keep pace with the needs of the target population.

## Supplemental Material

Supplementary_File_1 – Supplemental material for Mass media and communication interventions to increase HIV testing among gay and other men who have sex with men: Social marketing and visual design component analysis Supplemental material, Supplementary_File_1 for Mass media and communication interventions to increase HIV testing among gay and other men who have sex with men: Social marketing and visual design component analysis by Julie Riddell, Gemma Teal, Paul Flowers, Nicola Boydell, Nicky Coia and Lisa McDaid in Health:

Supplementary_file_2 – Supplemental material for Mass media and communication interventions to increase HIV testing among gay and other men who have sex with men: Social marketing and visual design component analysis Supplemental material, Supplementary_file_2 for Mass media and communication interventions to increase HIV testing among gay and other men who have sex with men: Social marketing and visual design component analysis by Julie Riddell, Gemma Teal, Paul Flowers, Nicola Boydell, Nicky Coia and Lisa McDaid in Health:

## Notes

1. Whilst the ‘United against Aids’ campaign was not focused on any one population, they used a variety of different characters to promote the message that ‘AIDs is everyone’s concern’. The study aimed to gather information regarding campaign awareness and impact in three sample groups; general population, MSM and migrants.

2. Image depicted an attractive male with tanned, sculpted muscles and unblemished skin, dressed only in white fitted underpants.
