# Supplementary material for: Mass media and communication interventions to increase HIV testing among gay and other men who have sex with men: Social marketing and visual design component analysis
Source: Health (London). 2020 Sep 19;26(3):338–60. doi: 10.1177/1363459320954237 (PMC8938994; doi:10.1177/1363459320954237)
Supplement: Supplementary_File_1 – Supplemental material for Mass media and communication interventions to increase HIV testing among gay and other men who have sex with men: Social marketing and visual design component analysis [file Supplementary_File_1.pdf]

Supplementary File 1: Search strategy example

Epub Ahead of Print, In-Process & Other Non-Indexed Citations, Ovid MEDLINE(R) Daily and Ovid MEDLINE(R) 1946 to Present 2.11.16

1	exp HIV Infections/	253789
2	exp HIV-1/	72027
3	exp Sexually Transmitted Diseases/	308146
4	Acquired Immunodeficiency Syndrome/pc [Prevention & Control]	13358
5	"human immunodeficiency virus".tw.	77759
6	HIV*.tw.	271457
7	"human immuno-deficiency virus".tw.	215
8	"acquired immune deficiency syndrome".tw.	5393
9	"acquired immuno-deficiency syndrome".tw.	116
10	"acquired immune-deficiency syndrome".tw.	5393
11	(human immun* and deficiency virus).mp	552
12	1 or 2 or 3 or 4 or 5 or 6 or 7 or 8 or 9 or 10 or 11	420489
13	exp Advertising as Topic/	13769
14	exp Cell Phones/	8099
15	exp Internet/	62577
16	exp "Marketing of Health Services"/	16714
17	exp Mass Media/	42237
18	exp Mobile Applications/	1435
19	exp Computers, Handheld/	3414
20	exp Smartphone/	609
21	exp Social Marketing/	2316
22	exp Social Media/	3158
23	exp Social Networking/	1779

24	"broadcast media".tw.	88
25	"cell phone".tw.	1331
26	"chat room".tw.	119
27	"digital literacy".tw.	50
28	"gay sexual networking".tw.	3
29	"mass communication".tw.	227
30	"mass media".tw.	4084
31	"new media".tw.	555
32	"mobile device".tw.	544
33	"mobile phone".tw.	3634
34	"personal digital assistant*".tw.	958
35	"sexual media".tw.	29
36	"smart phone".tw.	335
37	"social media".tw.	3802
38	"social networking applications".tw.	14
39	"text messag*".tw.	2166
40	App.tw.	16601
41	apps.tw.	2233
42	Facebook.tw.	1735
43	GSN.tw.	251
44	Internet.tw.	37642
45	Iphone.tw.	494
46	Online.tw.	65740
47	PDA.tw.	7526
48	Smartphone.tw.	2798
49	texting.tw.	477

50	Web.tw.	71401
51	Website.tw.	11939
52	Websites.tw.	6907
53	media.tw.	212557
54	13 or 14 or 15 or 16 or 17 or 18 or 19 or 20 or 21 or 22 or 23 or 24 or 25 or 26 or 27 or 28 or 29 or 30 or 31 or 32 or 33 or 34 or 35 or 36 or 37 or 38 or 39 or 40 or 41 or 42 or 43 or 44 or 45 or 46 or 47 or 48 or 49 or 50 or 51 or 52 or 53	490296
55	exp Homosexuality, Male/	12035
56	exp Bisexuality/	3432
57	exp Transsexualism/	3182
58	"cisgender male".tw.	8
59	"men who have sex with men".tw.	7634
60	"same sex".tw.	5334
61	"same-sex".tw.	5334
62	Bisexual.tw.	6129
63	Gay.tw.	8148
64	Homosexual*.tw.	12701
65	MSM.tw.	6762
66	Queer.tw.	648
67	transexual.tw.	16
68	transgender.tw.	2235
69	transsexual.tw.	1029
70	YMSM.tw.	215
71	55 or 56 or 57 or 58 or 59 or 60 or 61 or 62 or 63 or 64 or 65 or 66 or 67 or 68 or 69 or 70	39854
72	12 and 54 and 71	1569
73	limit 72 to yr="2009 -Current"	1143
