# Supplementary material for: Mass media and communication interventions to increase HIV testing among gay and other men who have sex with men: Social marketing and visual design component analysis
Source: Health (London). 2020 Sep 19;26(3):338–60. doi: 10.1177/1363459320954237 (PMC8938994; doi:10.1177/1363459320954237)
Supplement: Supplementary_file_2 – Supplemental material for Mass media and communication interventions to increase HIV testing among gay and other men who have sex with men: Social marketing and visual design component analysis [file Supplementary_file_2.pdf]

Supplementary table 1: Description of included interventions' social marketing mix

	Product							Place	Promotion	Price
**Reference**	**Intervention delivery by whom/what**	**Intervention content**	**Frequency & duration of individual interventions**	**Intensity of individual intervention(s)**	**Branding**	**Tone**	**Imagery**	**Intervention setting(s)/channel**	**Mode(s) of delivery**	**Costs/barriers that target users have to bear and barriers they have to overcome**
Gimmie 5 minutes **McOwan et al (2002), England, UK**	Delivered via adverts in free tabloid newspaper, posters in venues and take-away leaflets.	Campaign materials: (1) full-page advertisements in a free tabloid newspaper, (2) A4 posters in gay venues (3) leaflets in venues that could be removed. Content was same for both leaflet/ posters.	12 week campaign, newspaper images rotated on weekly basis	Unclear re length of time people would engage with materials	Campaign name, use of identified clinics	Primarily positive (about facilitating individual choice) however, content of materials describe pros and cons of HIV testing. Serious, direct, informal language.	Use of images of naked upper torso of MSM (1 young; 1 south European origin; 1 black origin) reflecting target audience. Very dense text section	Newspaper provided in gay friendly venues/ Gay venues in Central London	A4 poster, credit card sized leaflet in gay venues, full-page advertisement in free tabloid	not explicitly stated
Make your position clear **Flowers et al (2013) Glasgow, Scotland**	Posters and leaflets , outreach workers involved in distribution; online and smartphone app	Posters and leaflets, adverts were online and smartphone app. Promote sexual health behaviours and regular HIV testing	October 2009 to July 2010	Unclear re length of time people would engage with materials	Use of 'Make your position clear' and logos	Use of humour, informal, direct. Some sexually explicit	Six images were used: four were designed for display in gay scene and two for wider use. All images included two men, a 'position' name and number and one of the	Clinical, wider community, Gay scene venues and events, five online sites, smart phone application designed for MSM.	online, posters, leaflets, smartphone app	not explicitly stated

							key messages			
The HIV wake up campaign **Hilliam et al (2011) Scotland, UK**	leaflets and posters for MSM/ Health professionals, digital online banners, targeted web pages, emails targeting Gaydar users	leaflets and posters for MSM/ Health professionals, digital online banners, targeted web pages, emails targeting Gaydar users	Launched May 2010- no other information	Unclear re length of time people would engage with materials	Campaign name	not discussed and no images included	not discussed and no images included	online, Gaydar sites, scene venues, health related locations, wider community	leaflets, posters, digital online banners, targeted webpages/ direct emails	not explicitly stated
I did it **Hickson et al (2015) England, UK**	media advertisements, radio advert, website	media advertisements, radio advert, website	December 2010- April 2011	Unclear re length of time people would engage with materials	Campaign name	informative, positive	not discussed and no images included	radio/ online but not clear if gay scene specific or wider	radio, text/ media advertisements, online	not explicitly stated
**Brady et al (2014) England, UK**	Adverts, posters	Marketing HIV testing service on social media and through Grindr	January-September 2013	Unclear re length of time people would engage with materials	Unclear from paper, images suggest THT logos	Positive, , Direct, Informative- where to get tests	variety of imagery used: 1)text only 2) celebrity image and text 3) image of bed and text	Grindr adverts, no details of campaign settings	Grindr advert and poster specifically stated	ease of access to clinical services and fear of stigma.
I'm testing **James (2015), England, UK**	advertising, expanded testing services by stakeholders, campaign website, local delivery partners	targeted print, social media and outdoor advertising. A campaign website with information about testing services, risk assessments, clinic finder and free home sampling kits. Expanded testing services by stakeholders	four weeks	Unclear re length of time people would engage with materials	Use of logos and campaign slogan	Informative, direct to reader, responsibility focused. Positive (happy people)	All follow similar format, image of person with campaign slogans 'Im testing' logos and website information. Variety of people included in adverts (male and female, including openly gay celebrity doctor)	Wider community (e.g. tube/ buses/ phone boxes) and outside testing locations	print, social media and outdoor advertising, campaign website, testing events.	not explicitly stated

**West et al (2015) England, UK**	grindr advert/ posters	grindr advert with link to website containing video demonstrating HIV POCT. Posters	not discussed	Unclear re length of time people would engage with materials	video used clinic name	Factual, familiarise, direct, reassuring (e.g. re ease of testing)	Female shown getting tested for HIV using POCT, male doctor explains full process during video	Grindr, posters (unclear where), online video	Grindr advert, video on website, posters	not explicit in paper
United against AIDS **Prati et al (2016), Italy**	characters used in videos that audience were assumed to identify with, use of celebrity to deliver final message.	Television and radio public service announcements, print materials (e.g., posters, brochures), Web based advertisements, and cinema and newspaper advertisements.	4 weeks, 2 weeks each (December 2012/ February-March 2013)	Unclear re length of time people would engage with materials	Campaign name and use of ministry for health logo/ name	Positive-benefits/ advantages, identification with the characters. Serious	Different characters used to reflect wider populations, including male celebrity. Characters were draped in red ribbon, and looked to be naked underneath. Characters spoke direct to camera	wider community: mainstream media, cinemas, papers	television and radio public service announcements, print materials (e.g., posters, brochures), Web based advertisements, and cinema and newspaper advertisements.	not explicitly stated.
You know different' **Thackeray et al (2011) USA**	Community partners/ peer educators involved in distribution	Customisable posters, palmcards, stickers, Public service announcement scripts and tips to secure radio air time, and web ribbons to link with the campaign website. Face-to-face- card distribution	not discussed	Unclear re length of time people would engage with materials	Campaign name but unclear level of branding. Items customisable for specific clinic	Focus on responsibility/ self-respect	not discussed and no images included	not clear but suggests gay scene events etc., wider community in terms of radio/ posters	Customisable posters, palmcards, stickers, PSA (public service announcement) scripts and tips to secure radio air time, and web ribbons to link with the campaign website. Face-to-face in handing out cards	Denial of risk, fear, stigma, misinformation, and adult-oriented materials. **Price:** Risk of HIV and AIDS, judgment from health care system. Confirming status= stigma/ need to change behaviour

Talking about HIV **Hirshfield et al (2012) United States of America**	online documentary	5-minute documentary using HIV positive men discussing their experiences, footage came from longer documentary (Meth)	5-minutes, no record of if individual engaged/ watched full video	5 minutes for video, Unclear re length of time people would engage with materials	Campaign name, http://hivbigdeal.org,	Positive and negative modelling, use of emotion (personal experience) Serious.	Variety of HIV positive men talk to camera about personal experiences	online, also available through YouTube	online website/ YouTube	not explicitly stated
The morning after' **Hirshfield et al (2012) Chiasson et al (2014) United States of America**	banner ads that linked to drama based video	Nine minute drama that depicts 3 gay male friends, one of whom thinks he had unprotected sex with an HIV-positive man while intoxicated and seeks advice from friends.	9 minutes, no record of if individual engaged/ watched full video	video lasted 9 minutes, no record of if individual engaged/ watched full video	Intervention name 'The morning after', http://hivbigdeal.org,	Positive and negative modelling, use of emotion (drama). Use of fear, serious	Dramatic video where a gay male thinks he had unprotected sex with an HIV-positive man while intoxicated and seeks advice from friends.	Online, also available through YouTube	online via recruitment advert on exit page of one of the largest subscription-based gay sex sites	Not explicitly discussed
**Erausquin et al (2009) Los Angeles County, USA**	Trained outreach volunteers similar to target population distributed outreach cards, also advertised on internet and gay/bisexual orientated magazines	bilingual outreach cards with information about testing services; included voucher to be exchanged for movie pass.	August-October 2004	Unclear re length of time people would engage with materials	Not discussed and no images	not discussed and no images included	not discussed and no images included	Recruited at Latino-oriented gay scene events	face-to-face distributed outreach cards, internet advertisements, adverts in gay/ bisexual oriented magazines	Age, ethnicity, sexual behaviour, and lack of economic and social-environmental resources. May avoid tests because of fear of a positive HIV test result, lack of awareness re treatment resources, lack of knowledge of and access to health services that are gay-friendly, youth-friendly, and culturally sensitive. Experiences or

										fear of homophobia and racism
**Tu Amigo Pepe Solorio et al (2016), Seattle, USA**	avatar (pepe), posters, outreach, radio etc	Spanish-language radio PSAs, a Web site, social media outreach, a mobile based reminder system, print materials, and a free hotline.	4 weeks	Unclear re length of time people would engage with materials	Campaign name, character of Pepe	reframed negative attitudes, beliefs and norms towards positive ones about HIV testing, also used humour	Use of Pepe character/ peer videos	wider community: online/ radio/ posters	radio public service announcements, posters, website	limited access to HIV prevention information and HIV testing within the health care system, lack of health insurance, language barriers, self-identification and/ or disclosure as gay/ bisexual. Specific community based stigma around HIV/ sexual orientation.
Get Tested with Via Libre **Blas et al (2010) Lima, Peru**	banner ads that linked to drama based video	Two videos one targeted to gay identified MSM and one targeted to non-gay-identified MSM. The videos focused on ways to overcome variety of reasons why MSM don't get tested for HIV and transitioned through the stages of change. Emphasised free testing at clinic.	5 minutes, no record of length of video participant watched/ number of times.	5 minutes for video, Unclear re length of time people would engage with materials	Banner advertisement contained name of project (SOMOS), use of logos at end of video	Emotion (drama video), Serious, Initially fear then moves to reassurance, calm, informative at clinic. Positive (taking action),	Videos framed within Health Belief model and aimed to identify strategies to overcome reasons for not testing specific to target audience.	Online: five commercial gay sites, two advocacy websites. Videos also available on YouTube	Online via websites, YouTube	Fear of consequences of a positive test result; fear of discrimination; fear of non-confidentiality; fear of lack of support; knowledge about testing services; lack of monetary resources (e.g. to test/ pay for treatment where required) and perception of risk.

Hottest at the start **Gilbert et al (2013) British Columbia, Canada**	Posters, post cards, urinal ads, and condom packs at a variety of gay venues, e-mail blasts and campaign websites	Posters, post cards, urinal ads, and condom packs at a variety of gay venues, e-mail blasts and campaign websites	June-August 2011	Unclear re length of time people would engage with materials	Campaign name	very sexualised images, arousal, direct, informal	Images of men engaged in sexual activity, accompanied by text stating benefits of testing	gay venues, online	posters, leaflets, email blasts, online	Focus on benefits not costs
Check it out **Guy et al (2009) Victoria, Australia**	A4 posters and take away cards	A4 posters and take away cards	community attached MSM-over 5 months. non-community attached MSM and young MSM-over 6 weeks. non-community MSM from CALD backgrounds-over 6 weeks	Unclear re length of time people would engage with materials	Campaign name	Humour and informal	use of humour, e.g. two cockerels facing each other/ pair of male legs with trousers around ankles. Images matched tone of text.	Gay scene and wider community	A4-sized posters and takeaway cards. Printed advertisements, radio programmes online banner and a website.	not explicitly stated
**Drama down under Pedrana et al (2012) Wilkinson et al (2016) Victoria, Australia**	mainstream advertising, community based advertising	print and radio advertisement, outdoor advertisements, public events, and online banners, 'novel' campaign resources (e.g., fridge magnets, drink holders, and underwear) and campaign-specific events (e.g., the "Drama Down Underwear" Show). Materials included images and brief messages aimed	Phase I (February 2008–February 2009), Phase II (March 2009–May 2010); Phase III (June 2010–June 2011)	Unclear re length of time people would engage with materials	Campaign name/ logos	light-hearted, informative, direct, humour, informal language	Variety of imagery depicting same male in different scenarios, each image included text relating to campaign message	wider community, online, gay scene	print and radio advertisement, outdoor advertisements, public events, and online banners, 'novel' campaign resources (e.g., fridge magnets, drink holders, and underwear) and campaign-specific events (e.g., the "Drama Down Underwear" Show).	not explicitly stated.

		at target audience, included referral to campaign Website for more information.								
Crowdfunding video **Tang et al (2016) China**	dramatic video	One minute online video showing 2 men initiating relationship and testing for HIV together	1 minute	1 minute long, suggested that they were able to monitor single/ multi-views but not clear how.	use of logos in video end screens	positive/ romantic (togetherness), informative, aspirational, serious, responsibility	Variety of scenarios suggesting storyline of 2 Chinese men falling in love and testing for HIV together.	online, although part of competition so wider community?	video, available on YouTube	not explicitly stated
Health marketing video **Tang et al (2016) China**	cartoon video depicting character performing risky behaviours	1 minute online video used a cartoon storyline to provide HIV education and promoting HIV testing.	1 minute	1 minute long, suggested that they were able to monitor single/ multi-views but not clear how.	use of logos in video end screens	informative, use of humour/ stereotypes, childlike, informal	Cartoon storyline following main character engaging in risky behaviour and finally testing for HIV.	online	video, online	not explicitly stated

Supplementary table 2: Reading the Visual

Campaign name	Reference	Actor's Appearance (Actor = any people in the visual)			Setting/ Environment	Props/Objects	Form of representation (narrative OR conceptual)[a]	Contact - Demand or Offer	Social Distance[b]	Point of view: Engagement[c]	Point of View: Power[d]	Compositional (Salience)[e]	Modality[f]
**Make Your Position Clear**	Flowers et al (2013)	Actors Explicitly MSM	Naked, Clothed	Target Audience - Attractive	Neutral = Focus On Actors	Various To Communicate An Environment To Suit The Pun	Narrative - Transactional	Offer	Social	Detachment (One Character In One Poster Involvement)	Equality	Actors	Low - Staged In Studio
**Gimmie 5 minutes**	McOwan et al (2002)	Actors Not Explicitly MSM	Naked	Target Audience - Normal/ Attractive	Neutral = Focus On Actors, Bright Colour = Attract Attention	None	Narrative - Vector Is Completed By Eye Contact With Viewer	Demand	Social	Involvement	Equality	Actors and Text	Low - Staged In Studio
**Hottest at the start**	Gilbert et al (2013)	Actors Explicitly MSM - Stereotyped Appearance Of Gay Men	Naked, Clothed	Target Audience - Attractive	Range Of Everyday Public Locations (Work, Kitchen, Gym) = Sex In Public Space = Sexually Promiscuous	Tattoos/ Piercings = Fashionable/Edgy?	Narrative - Transactional	Offer	Social	Detachment (One Character In One Poster Involvement)	Equality	Actors	Medium - Staged In Real Setting
**Health Marketing video**	Tang et al (2016)	Actors = Range Of Characters, Central Character Not MSM	Not Naked	N/A	Cartoon Street	Umbrella = Condom	N/A	Offer	Impersonal	Detachment	Equality	Actors	Low - Cartoon
**Crowdsourcing video**		Actors Explicitly MSM	Not Naked	Target Audience - Attractive	Real Restaurants, Shops, Clinic Etc	Books = Intelligent, Red Ribbon = HIV, Results = Testing	N/A	Offer	Social	Both	Equality	Actors	Medium - Staged In Real Setting

**Variety of campaign images**	Brady et al (2014)	1) Bed Viewed From Above, 2) Text Only, 3) Actor Not Explicitly MSM	Not Naked	3) = Target Audience - Attractive	1) Bed Symbolising Sex	N/A	1) Narrative (Vector In Displacement Of Sheets) 2) Conceptual, 3) Narrative - Vector Is Completed By Eye Contact With Viewer	1) & 2) Offer, 3) Demand	3) Social	3) Involvement	3) Equality	1) Bed 2) Text 3) Actor	Medium/ N/A
**I'm testing**	James (2015)	1) Celebrity Who Is Gay, 2) Actors Not Explicitly MSM	Not Naked	All Attractive	Neutral = Focus On Actors, Bright Colour = Attract Attention	Stethoscope = Doctor	Narrative - Vector Is Completed By Eye Contact With Viewer	Demand	Social	Involvement	Equality	Actors	Low - Staged In Studio
**Get Tested with Via Libre -non-gay identified video**	Blas et al. (2010)	Actors Explicitly MSM Via Narrative	Not Naked	Target Audience - Normal	Realistic Home (Poor Neighbourhood), Real Clinic	Familiarise With Clinic, Test Results, Testing	N/A	Both	All	Both	Equality, Viewer Power (opening scene)	Actors	Setting = High, Acting = Dramatised?
**Get Tested with Via Libre -gay identified video**	Blas et al. (2010)	Actors Explicitly MSM Via Narrative	Not Naked	Target Audience - Normal	Realistic Home (Wealthy Neighbourhood), Real Clinic	Familiarise With Clinic Testing, Test Results, Testing	Narrative	Both	All	Both	Equality	Actors	Setting = High, Acting = Dramatised?
**United against Aids**	Prati et al (2016)	1) Celebrity (Unknown To Me), Range Of Actors M/F	Naked	Not Target Audience - All Attractive	Neutral = Focus On Actors	Red Ribbon = HIV	N/A	Demand	Social	Involvement	Equality	Red ribbon	Low - Staged In Studio
**POCT video**	West et al (2015)	Actor 1 = Clinician, Actor 2 = Older Female	Not Naked	Not Target Audience	Real Clinic	Familiarise With Clinic, Test Results, Testing	N/A	Both	Social	Detachment	Equality	Actors	High - Filmed Real Testing In Real Setting With Real People (Homemade Feel To Video Quality)

**The Morning after**	Chiasson et al (2014) [g]	Actors Explicitly Msm Via Narrative	Naked In Appropriate Scenes	Target Audience - Attractive	Realistic, Home, Cafes, Streets, Clinic	Laptop/Dating Website = Hooking Up With Strangers?	N/A	Offer	Social	Both	Equality	Actors	Medium - Staged In Real Setting
**Check it out**	Guy et al (2009)	1) Cockerels, 2) Trousers Around Ankles (Male)	1) N/A, 2) Semi Naked	Not Target Audience	None, Wooden Floorboards	Cockerels = Male, 'Cock', Fighting Stance = Confrontational?	Narrative	Offer	Intimate close up of trousers?	N/A	N/A	Image	Low - Staged In Studio; Medium - Staged In A Normal Environment
**Talking about HIV**	Hirshfield et al (2012)	Real People, Explicitly MSM	Not Naked	Target Audience - Normal	Private, Intimate Settings For Interview? = Intimacy	Laptop/Dating Website = Hooking Up With Strangers?	N/A	Demand	Intimate- Head shots	Involvement	Equality	N/A	High - Real People Not Actors, Real Experiences
**Drama down under**	Wilkinson et al (2016)	Actor Not Explicitly MSM (Same Throughout), But Inferred - Shaved Chest, Underwear, Camp Expressions	Semi Naked	Target Audience - Attractive	Neutral = Focus On Actors	Underwear - Tighty Whities = Highlight Genitals? Confidence? Humour? Plus Various Props: Animals, Seasons, To Suit Pun/Time Of Year Of Campaign	Narrative - Vector Is Completed By Eye Contact With Viewer	Demand	Social	Involvement	Equality	Actor	Low - Staged In Studio
	Pedrana et al (2012)	Actor Not Explicitly MSM (Same Throughout), But Inferred - Shaved Chest, Underwear, Camp Expressions	Semi Naked	Target Audience - Attractive	Neutral = Focus On Actors	Underwear - Tighty Whities = Highlight Genitals? Confidence? Humour? Plus Various Props: Animals, Seasons, To Suit Pun/Time Of Year Of Campaign	Narrative - Vector Is Completed By Eye Contact With Viewer	Demand	Social	Involvement	Equality	Actor	Low - Staged In Studio

[a] Literature distinguishes between the two in terms of Narrative having a VECTOR - a line (often diagonal) that connects actors within an image or an arrow connecting boxes in a diagram. The other actor could be out of shot - but if the actor is looking or pointing/reaching at someone or something then there is a vector.

[b]In terms of: Intimate (close up shot, e.g. headshot of actor) Social (Medium e.g. actor is as close as you would normally get to a person in a friendly social situation) OR Impersonal (distance shot of people, e.g. strangers in public)

[c] Engagement in terms of: Involvement (actor facing viewer/front on Detachment (actor in profile/side on)

[d] Power in terms of:Viewer power (top down view on actors) Equality (eye level with actors) OR Representation power (looking up at actors from below)

[e] What is highlighted as importance and why?

[f] How true to real life is the image, level of 'truth' represented - High/Medium/Low

[g] note campaign also used by Hirshfield et al, 2012.
